# Hyperbaric Oxygen Prevents Early Death Caused by Experimental Cerebral Malaria

**DOI:** 10.1371/journal.pone.0003126

**Published:** 2008-09-04

**Authors:** Yara C. Blanco, Alessandro S. Farias, Uta Goelnitz, Stefanie C. P. Lopes, Wagner W. Arrais-Silva, Bruna O. Carvalho, Rogério Amino, Gerhard Wunderlich, Leonilda M. B. Santos, Selma Giorgio, Fabio T. M. Costa

**Affiliations:** 1 Department of Microbiology & Immunology, State University of Campinas – UNICAMP, Campinas, São Paulo, Brazil; 2 Department of Parasitology, UNICAMP, State University of Campinas, Campinas, São Paulo, Brazil; 3 Department of Parasitology – ICB, University of São Paulo – USP, São Paulo, São Paulo, Brazil; 4 Department of Biochemistry, Federal University of São Paulo – UNIFESP, São Paulo, São Paulo, Brazil; Federal University of São Paulo, Brazil

## Abstract

**Background:**

Cerebral malaria (CM) is a syndrome characterized by neurological signs, seizures and coma. Despite the fact that CM presents similarities with cerebral stroke, few studies have focused on new supportive therapies for the disease. Hyperbaric oxygen (HBO) therapy has been successfully used in patients with numerous brain disorders such as stroke, migraine and atherosclerosis.

**Methodology/Principal Findings:**

C57BL/6 mice infected with *Plasmodium berghei* ANKA (PbA) were exposed to daily doses of HBO (100% O_2_, 3.0 ATA, 1–2 h per day) in conditions well-tolerated by humans and animals, before or after parasite establishment. Cumulative survival analyses demonstrated that HBO therapy protected 50% of PbA-infected mice and delayed CM-specific neurological signs when administrated after patent parasitemia. Pressurized oxygen therapy reduced peripheral parasitemia, expression of TNF-α, IFN-γ and IL-10 mRNA levels and percentage of γδ and αβ CD4^+^ and CD8^+^ T lymphocytes sequestered in mice brains, thus resulting in a reduction of blood-brain barrier (BBB) dysfunction and hypothermia.

**Conclusions/Significance:**

The data presented here is the first indication that HBO treatment could be used as supportive therapy, perhaps in association with neuroprotective drugs, to prevent CM clinical outcomes, including death.

## Introduction

Cerebral malaria (CM) causes 1–2 million deaths annually; mainly in sub-Saharan African children aged 2–6. It is estimated that 250,000 children that do not succumb to CM will develop neurocognitive impairments per year [1] and most CM patients die before the beneficial effects of drug treatment are observed [2]; thus indicating the need to explore new supportive therapies.

CM is a multi-factorial syndrome characterized by neurological signs, seizures and coma, which can, in turn, lead to death. This syndrome can be associated with a loss of cerebrospinal fluid spaces and ischemia [3], alterations in cerebral blood flow velocity [4], a decrease in cerebral oxygen consumption in CM comatose patients [5] and an increase in the lactate levels of the cerebrospinal fluid [6] which decreases after patients recover consciousness [7]. Recent imaging and postmortem analyses have revealed the presence of Durck granulomas, blood-brain barrier (BBB) dysfunction and diffuse cerebral edema with multiple petechial hemorrhages and ischemic changes in the brain of adults with CM [8], [9].

Although the CM pathogenic process is controversial and still not fully understood, evidence suggests that the host's immune system plays a major role in expressing certain cytokines, e.g. TNF-α and IFN-γ, and activating immunocompetent cells [10]–[15]. In fact, recent immunological analyses have shown that, unlike individuals with mild and severe non-cerebral malaria, CM patients present elevated levels of a specific cluster of cytokines, which include TGF-β, TNF-α, IL-1β and IL-10 [16].

Hyperbaric oxygen therapy (HBO; pO_2_ = 760 mmHg) has been successfully used against bacterial and fungal infections and as an adjunct therapy in surgeries [17]–[19]. In addition, reports have recently shown that HBO therapy transiently suppresses the inflammatory process of ischemic wounding and trauma [20], [21]. Indeed, immunological analyses have revealed that HBO therapy significantly decreases the levels of TNF-α and IL-1β secreted by monocytes and macrophage collected from rats or from human peripheral blood after stimulation with LPS [22], [23]. In an experimental model for ischemia, HBO reduces immunocompetent cell sequestration and the synthesis of TNF-α [24]; probably by decreasing ICAM-1 expression levels [25]. Moreover, HBO reduces the expression of the cyclooxygenase-2 (COX-2) mRNA, an enzyme involved in inflammation, and the hypoxia-inducible factor-1α (HIF-1α), a transcriptional factor associated with low oxygen concentrations [26], [27]. HBO therapy has been used in patients with numerous brain disorders such as stroke, migraine and atherosclerosis, due to its capacity to decrease cerebral edema and brain infarction while maintaining BBB integrity, reducing neuronal death and improving blood flow in damaged areas of the brain [28]. Nevertheless, depending on the protocol used for treatment, HBO therapy has potential side effects associated to ear and sinus barotraumas, myopia and convulsion [29].

In an early study, HBO was observed to alter the parasitemia levels of mice infected with a non-cerebral line of *Plasmodium berghei* [30]. However, the HBO effect on the entire curve of parasitemia, on the clinical symptoms and on the mechanisms of the illness were not further investigated. Moreover, although the pathological process involved in CM displays some features in common with brain stroke, the effect of HBO on CM, to our knowledge, has never been assessed. Here we show that in conditions also suitable for human use, HBO therapy prevents CM clinical symptoms in C57BL/6 mice infected with *P. berghei* ANKA, a model widely used for experimental cerebral malaria (ECM) [31].

## Methods

### Mice and parasites

C57BL/6 mice (7–10 weeks old) were purchased from the University of São Paulo (São Paulo, SP, Brazil) and maintained in our specific pathogen-free animal facility. All experiments and procedures were approved by the UNICAMP Committee for Ethics in Animal Research (Protocol No. 857-1).

Two different strains of *P. berghei* were used: the cloned line of *P. berghei* ANKA (PbA) and *P. berghei* NK-65 (PbNK-65), respectively an ECM- and non-ECM-causing strain; kindly provided by Dr. Laurent Rénia (Singapore Immunology Network, Agency for Science, Technology and Research, Biopolis, Singapore) and Dr. Nobuko Yoshida (Federal University of São Paulo, São Paulo, SP, Brazil), respectively. The blood stage forms of both parasites were stored in liquid nitrogen after *in vivo* passages in C57BL/6 mice according to the protocol described elsewhere [31]. Mice were infected intraperitoneally (i.p.) with 10^6^ infected red blood cells (iRBC) and parasitemia and the neurological signs for CM were monitored daily.

### Hyperbaric oxygen treatment

Groups of 8–10 PbA-infected mice were exposed daily to 100% oxygen at a pressure of 3.0 atmospheres (ATA) for 1 h per day in a hyperbaric animal research chamber (Research Chamber, model HB 1300B, Sechrist, USA) from day 0 to 10 post-infection (11-day exposure), or for 2 h from day 4–7 post-infection (4-day exposure). The chamber was pressurized and decompressed at a rate of 0.5 ATA/min as described elsewhere [32]. For the 11-day exposure protocol, mice were previously exposed to HBO for 1 h before PbA infection, whereas for the 4-day exposure protocol, PbA-infected mice were randomly selected and placed in the hyperbaric chamber. To determine the effect of 100% oxygen (hyperoxia), regardless of pressurization, PbA-infected mice were submitted to the 11-day exposure protocol, but at 1.0 ATA (normobaric) instead of 3.0 ATA. Infected mice in the control group (non-exposed) were left in an airy room. The temperature inside the hyperbaric chamber was 21°C, the same as in the room, and was measured with the aid of a high-pressure resistant thermometer (model TB-0261, Instrucamp, Brazil). For the direct HBO effect assays, normal red blood cells (nRBC) or iRBC were collected from a naïve mouse or a PbA-infected animal on day 6 post-infection (12% parasitemia), and then diluted in an RPMI 1640 medium (Sigma, USA) supplemented with 10% of fetal bovine serum (Hyclone, USA). One mL of nRBC or iRBC (10^7^/mL) were plated in five replicates on a 24 well-plate and exposed to HBO (100% O_2_, 3.0 ATA) in a hyperbaric chamber for up to 6 hours.

### Parasitemia, temperature and red blood cell density assessment

The percentage of parasitemia was determined by counting the number of iRBC in at least 1,000 erythrocytes in Giemsa-stained blood smears. The mice's corporal temperature and the density of red blood cells (DRBC/mL×10^9^) were evaluated daily, starting on day −1 post-infection (p.i.), by rectal introduction of a precision digital thermometer (model TE-300, Instrucamp, Brazil), and with the aid of a Neubauer chamber, respectively. In the *in vitro* assays, DRBC were counted from 0 hour. The percentage of RBC density relative to day −1 p.i. or to 0 hour was calculated with the following formula: [(DRBC per mL×10^9^ of a determined day p.i. or hour/DRBC per mL×10^9^ on day −1 p.i. or at 0 h)×100].

### Measuring cytokine gene expression in the brain

The expression of several cytokine genes was evaluated by real-time quantitative reserve transcription-PCR (RT-qPCR) in the brain of PbA-infected animals removed on day 7 p.i.. Mice brains were frozen with crushed liquid nitrogen placed in the Trizol™ reagent (Invitrogen, USA) according to the protocol described by the manufacturer. Shortly, after the addition of 1 mL of Trizol™ (Invitrogen, USA) in 40 mg of the brain powder, 0.2 mL of chloroform was added and the lysate was vigorously mixed. The sample was centrifuged at 12,000× g for 15 min and the aqueous phase was transferred to a new tube. The RNA was precipitated by adding 0.5 mL of isopropanol followed by a centrifugation at 12,000× g, then washed with 1 mL of 75% ethanol and resuspended in RNAse free water. RNA was then treated with Deoxyribonuclease I (Fermentas, Canada) in order to degrade contaminating genomic DNA. The cDNA was synthesized using approximately 2 µg of the total RNA with the aid of the High Capacity cDNA Reverse Transcription Kit (Applied Biosystems, USA) according to the protocol provided by the manufacturer. The polymerase chain reaction was performed with an ABI Prism 7500 (Applied Biosystems, USA) and the reactions were carried out in 25 µL volume and in the presence of the TaqMan PCR Master Mix™ (Applied Biosystems, USA) and different sets of oligonucleotides and probes for the amplification of the β-actin, IFN-γ, TNF-α, IL-1β, IL-6 and IL-10 genes. These corresponded (respectively) to the following reference numbers (Applied Biosystems, USA): Rn00667869_m1, Mm00443258_m1, Mm00443285_m1, Mm00434228_m1, Mm00446190_m1 and Mm00439616_m1. Expression levels of cytokine genes in PbA-infected animals were represented as a relative copy numbers by using the delta threshold cycle method (2^−ΔCt^) [33].

### Purification of brain-sequestered T cells (BST)

Adherent leukocytes were isolated from mice brains as described elsewhere [14]. Briefly, on day 7 p.i., PbA-infected mice were perfused intracardially with PBS to remove both circulating and non-adherent RBC and leukocytes. Brains were collected and crushed in an RPMI-1640 medium (Sigma, USA) supplemented with 10% fetal bovine serum (Hyclone, USA) and gentamycin. The cellular suspension was collected and centrifuged at 15,000× g for 5 min. The pellet was resuspended with 10 mL of an HEPES buffer (Sigma, USA) and supplemented with collagenase (Roche, USA) and DNase I (Roche, Germany). The mixture was stirred at room temperature for 30 min. The tissue extract was passed through sterile gauze and centrifuged at 5,000× g for 30 s to remove debris. The supernatant was deposited on a 30% Percoll™ (GE Healthcare, Sweden) gradient and centrifuged at 3,000× g for 10 min. The pellet was collected and residual RBC were removed by an ACK lysis buffer. BST were resuspended in PBS containing 5% FBS and counted.

### Immunolabeling and flow cytometry analysis of BST

Cells were stained with appropriate dilutions of the following fluorochrome-labeled monoclonal antibodies (mAbs): FITC/anti-CD4 (clone H129-19), FITC/anti-CD8 (clone 53-6.7), PE/anti-TCR γδ (clone GL3) and APC/anti TCR αβ (clone H57-597) and then washed with PBS, fixed and analyzed by flow cytometry in a FACSCanto™ device (Becton Dickinson, USA). All these reagents were purchased from Pharmingen/Becton-Dickinson (USA). Analyses were performed after recording 10,000 events for each sample using Diva™ software. BST were identified by their size (forward light scatter) and granulosity (side light scatter) as previously described [34].

### Evaluating Blood-brain barrier dysfunction

Blood-brain barrier (BBB) integrity was assessed in PbA-infected mice on day 7 p.i. by i.v. injection of Evans Blue (1% in saline) in the retro-orbital plexus as previously described [35]. One hour after injection, mice brains were extracted and photographed using a digital camera (Nikon, USA). Brain staining was quantified by measuring the brightness intensity using the red channel in a delimited circular area of 12,294 pixels^2^ with the aid of the ImageJ™ software (http://rsb.info.nih.gov/ij). The brightness intensity of mice brain was inversely proportional to the levels of Evans Blue staining.

### Statistical analysis

The statistical significance between control and experimental groups were determined with the Log-Rank test for the cumulative survival experiments. The Mann-Whitney *U* test was used to compare parasitemia levels, the drop in relative temperature, the relative RBC density, BBB integrity and parasite and cytokine gene expression among brains collected from both naïve animals and infected mice. Calculations were performed using BioEstat™ version 3.0 (CNPq, Brazil) and Prism™ version 3.02 (Graphpad, USA) software. Values were considered significant when *P*<0.05.

## Results

### HBO effects on ECM associated mortality and on parasite development

**Figure 1:**
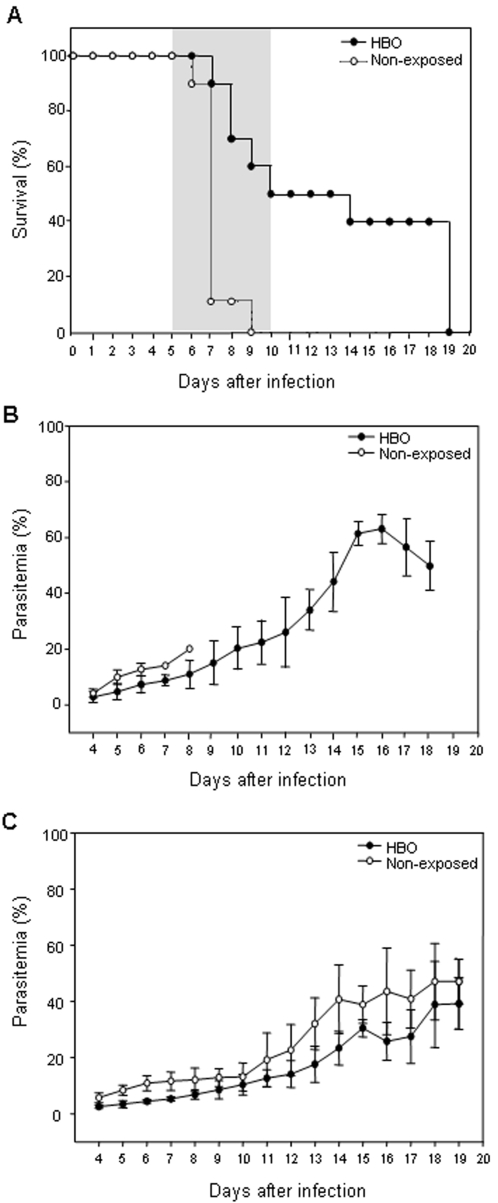
HBO's effect on the survival and the parasite development in *P. berghei*-infected mice. (A) Groups of 10 mice infected i.p. with 10^6^ iRBC were exposed or not to HBO (100% O_2_, 3.0 ATA) for 1 h from day 0 to 10. Pressurized oxygen significantly protected mice against CM neurological symptoms (*P*<0.0005). Neurological signs of CM appeared on days 5–10 with death occurring approximately 24 h after onset (shaded area). Parasitemia levels were assessed daily in mice infected with (B) *P. berghei* ANKA (PbA; cerebral line) or (C) *P. berghei* NK-65 (PbNK-65; non-cerebral line) regardless of exposure to HBO. HBO significantly (*P*<0.05) reduced the parasite burden on days 4–6 and 4–13 p.i., respectively in PbA- and PbNK-65-infected mice when compared to non-exposed animals.

To evaluate the neuroprotective effect of pressurized oxygen, two groups of 10 mice each were infected with PbA. One of these groups was submitted daily to HBO conditions (100% O_2_, 3.0 ATA, 1 hour) during 11 consecutive days. As shown on Figure 1A, 100% of PbA-infected mice not exposed to HBO exhibited CM-specific neurological signs within 5 to 8 days after infection and died of fatal cerebral malaria in the following 24 hours; most (80%) died on day 7 p.i.. All animals from this group were dead by day 9 p.i.. In contrast to the non-exposed animals, 50% of the mice from the HBO group did not develop CM symptoms and survived. In the HBO group, CM neurological signs began to appear later and the mortality rate increased slowly throughout days 7–10, representing 10, 20, 10 and 10%, respectively, on days 7–10. Of note, 1 animal (10%) died on day 14 and 4 (40%) on day 19 post-infection. Cumulative survival statistical analyses clearly demonstrated that HBO therapy had a significant (*P*<0.0005) neuroprotective effect against ECM. As expected, in the mice that did not develop CM, parasite burden progressed and mice died as a result of hyperparasitemia (Figure 1B).

As previously reported, HBO therapy inhibits the development of *Leishmania amazonensis* and of a non-cerebral line of *P. berghei* [30], [32], [36]. To further explore the effects of HBO, we monitored the parasitemia levels of infected mice exposed daily, or not, to HBO (11-day exposure protocol) for up to 19 days. We observed that HBO significantly (*P*<0.05) reduced the parasite burden of PbA-infected mice on days 4, 5 and 6 p.i., when compared to non-exposed animals (Figure 1B). However, since 100% of non-exposed PbA-infected mice died, we decided to evaluate whether the reduction on parasitemia levels in HBO exposed animals could be sustained over longer periods. Mice infected with *P. berghei* NK-65, a non-cerebral strain that displays similar parasitemia levels, were submitted to pressurized oxygen sessions as in the 11-day exposure protocol (Figure 1C). As observed in PbA-infected animals submitted to pressurized oxygen, a significant (*P*<0.05) decrease in PbNK-65 development was observed on day 4–13 p.i.. Nevertheless, no correlation was found between mice that presented a reduction of parasitemia levels with protection or attenuation of the neurological symptoms (Table S1).

**Figure 2:**
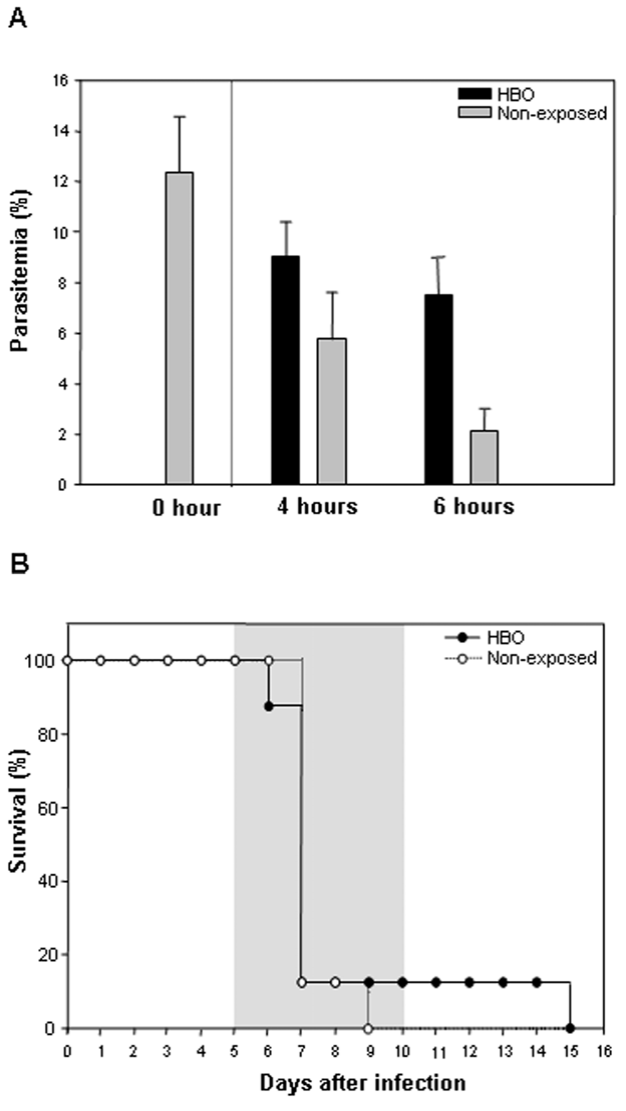
The direct effect of HBO therapy on RBC infected, or not, by PbA. 10^6^ iRBC/mL of PbA in a 24-well-plate were directly exposed or not to HBO (100% O_2_, 3 ATA). (A) Parasitemia levels were evaluated four or six hours after direct iRBC exposure to pressurized oxygen conditions. The parasite burden decreased significantly (*P*<0.05) after 4 or 6 hours in comparison to 0 hour. The reduction of parasitemia levels were more pronounced in infected cells left in normal room air than iRBC submitted directly to HBO after 4 (*P*>0.05) or 6 hour-exposure (*P* = 0.01). Results are expressed as the mean of quadruplicates±standard deviation. (B) Mice (n = 8 each group) were infected with 10^6^ iRBC of PbA collected after either six hours of direct exposure or no exposure to HBO. No statistical difference was noted when survival curves were compared (*P*>0.05).

Because we observed that HBO had a significant effect on the parasite burden in the infections of PbA and PbNK-65, we addressed the question as to whether pressurized oxygen therapy could damage normal red blood cells (nRBC) or inhibit parasite development directly. For this purpose, normal RBC (nRBC) collected from a naïve mouse were exposed to pressurized oxygen (100% O_2_, 3 ATA) during 4 or 6 hours. The relative percentage of nRBC density was not significantly altered (*P*>0.05) after direct exposure to HBO for up to 6 hours (data not shown), demonstrating that HBO therapy was not toxic to healthy erythrocytes in these conditions. Next, to evaluate HBO's effect directly on parasite development, infected RBC (iRBC) from a PbA-infected mouse were collected and exposed to HBO (100% O_2_, 3 ATA). Figure 2A shows a significant reduction (*P*<0.05) on parasite development after 4 and 6 hours in comparison to 0 hour, regardless of exposure to pressurized oxygen. However, when we compared the reduction on parasitemia levels of iRBC left in room air or exposed to HBO, we noticed a significant (*P* = 0.01) and more pronounced reduction of the non-exposed iRBC than of the infected cells directly exposed to HBO up to 6 hours. Inhibition of parasite development was also observed after 4 hours of exposure; however, no statistical difference was found (*P*>0.05). Then, to assess whether these iRBC were still able to induce CM neurological signs, we collected 10^6^ iRBC exposed directly to HBO or left outside the hyperbaric chamber for 6 hours and injected them in susceptible mice. As shown on Figure 2B, mice infected with iRBC directly exposed to HBO or with the cells left outside the chamber did not present significant differences (*P*>0.05) when the survival curves were compared. Taken together, these data suggest that 6 hours of HBO exposure do not directly affect PbA-infected erythrocytes nor alter their ability to induce CM clinical symptoms.

**Figure 3:**
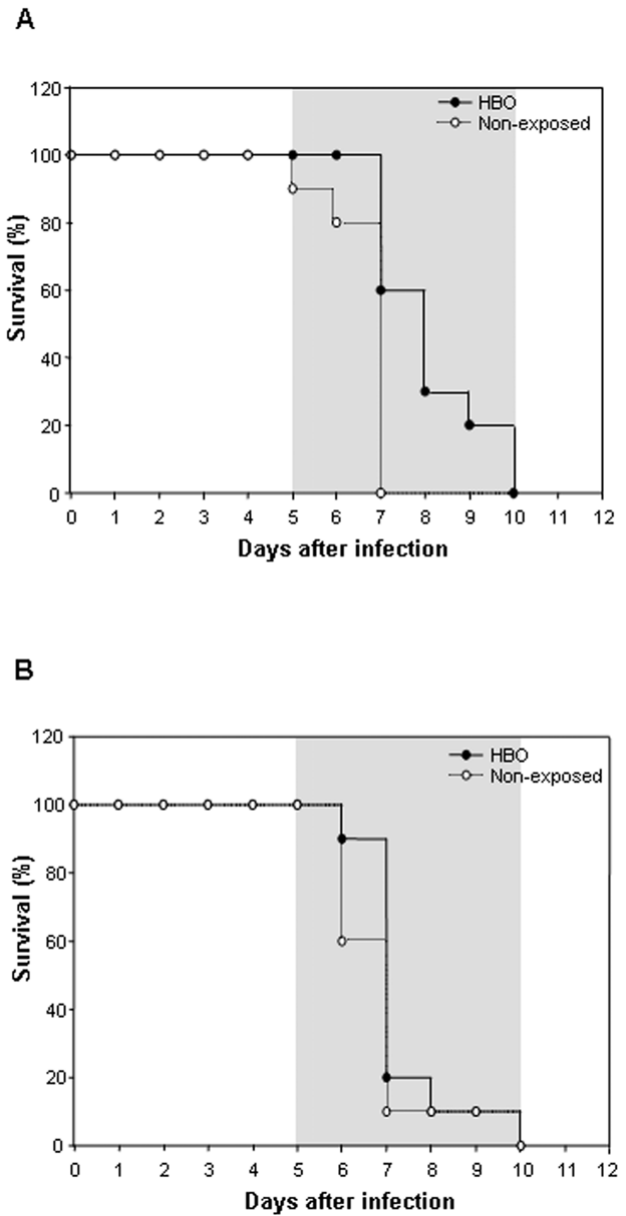
Evaluation of HBO's neuroprotective effect after parasite establishment and the role of pressure in mice survival. (A) Twenty mice were injected i.p. with 10^6^ iRBC; on day 4 p.i. (parasitemia of 4%) 10 animals, randomly selected, were daily exposed to HBO therapy (100% O_2_, 3.0 ATA) for 2 hours from days 4–7 after parasite inoculation. The survival curves of both groups demonstrated that HBO significantly delayed mice mortality (*P*<0.01). (B) Groups of 10 PbA-infected mice were exposed daily or not exposed to HBO (100% O_2_, 1 hour per day) at 1.0 ATA until all the animals died. Survival curves of the one hundred percent normobaric oxygen exposed mice and animals exposed to normal air did not differ significantly (*P*>0.05).

Next, to investigate whether pressurized oxygen could have an effect when parasitemia was already patent (4%), we randomly selected half of the PbA-infected mice on day 4 p.i. and exposed them to daily HBO sessions (100% O_2_, 3.0 ATA, 2 hours per day) until day 7 (Figure 3A). As expected, non-treated mice started to display CM clinical features early on day 5 and 6 and began dying within 20–24 hours on days 5 (10%) and 6 (10%), though the majority (80%) died on day 7 p.i.. All mice were dead by day 7. Notably, hyperbaric oxygen significantly delayed (*P*<0.01) CM specific mortality by up to two days, when compared to non-exposed animals, and reduced the rate of mortality on day 7 from 80% to 40% (Figure 3A). Moreover, two HBO-exposed mice (20%) only exhibited CM neurological signs on days 8 and 9, dying within 24 hours on days 9 and 10. This shows that HBO is capable of interfering significantly with the manifestation of the CM clinical symptoms, including death, even when administrated after parasite establishment. As observed in the 11-day exposure protocol, the administration of pressurized oxygen starting on day 4 p.i. (4-day-exposure) in PbA-infected mice reduced the parasitemia levels (*P*<0.01) significantly on days 4–6 (data not shown).

To confirm that only pressurized oxygen had neuroprotective effects, PbA-infected mice were submitted to the 11-day exposure protocol, but using 1.0 ATA as the atmospheric air pressure (Figure 3B). In this assay, no significant difference (*P*>0.05) was observed after cumulative survival analyses between infected animals exposed to HBO-1.0 ATA and the control mice. Of note, most of the non-exposed mice began to present CM symptoms and died earlier than the HBO-1.0 ATA treated animals. Although a minimal beneficial effect was observed after the administration of 100% oxygen (hyperoxia) under normobaric conditions, this was not enough to protect or even delay CM neurological symptoms, thus demonstrating that HBO's neuroprotective effect does not rely solely on the administration of 100% oxygen.

### The effect of HBO on cytokine expression levels and adherent T cells in the brain

**Figure 4:**
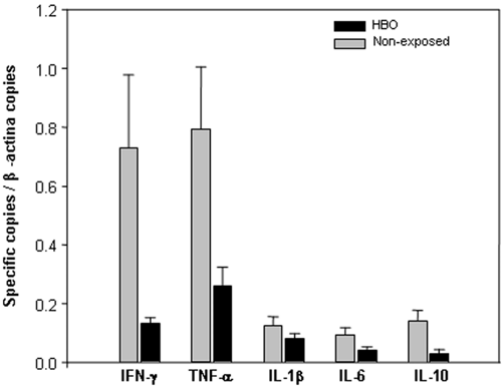
Cytokine gene expression is altered in the brains of PbA-infected mice exposed to HBO. Groups of 6–7 PbA-infected mice were either submitted or not to pressurized oxygen therapy (100% O_2_, 3.0 ATA, 1 hour per day) and on day 7 p.i. brains were collected for real-time quantitative reserve transcription-PCR analysis. HBO significantly reduced IFN-γ (*P*<0.05), TNF-α (*P*<0.01) and IL-10 (*P*<0.05), but did not alter IL-1β and IL-6 mRNA expression levels in contrast to non-exposed mice. Values are expressed as the mean of specific cytokine genes copies relative to β–actin copies of six-seven mice±standard deviation.

Based on the anti-inflammatory features of the HBO treatment reported in ischemic models [21], [26] and since the up-regulation of pro-inflammatory cytokines (IFN-γ, TNF-α and IL-1β) [10]–[12] and the participation of CD4^+^ and CD8^+^ T lymphocytes [14], [37] is essential for CM pathology to occur, we examined the mRNA levels of different cytokines in the brain of PbA-infected mice scarified on day 7 p.i.. According to Figure 4, after RT-qPCR analysis the mRNA levels of IFN-γ (*P*<0.05), TNF-α (*P*<0.01) and IL-10 (*P*<0.05) significantly decreased in the brain of mice submitted to the 11-day exposure HBO protocol in comparison to non-exposed animals. No significant difference (*P*>0.05) was noted in the mRNA levels of IL-1β and IL-6. RT-negative controls did not generate a detectable amplification product. All cDNA samples resulted in a product when the β-actin set of oligonucleotides and specific probe were present. Regardless of exposure to HBO, animals that presented an increase in the expression of IFN-γ mRNA also presented elevated levels of TNF-α and IL-10.

**Figure 5:**
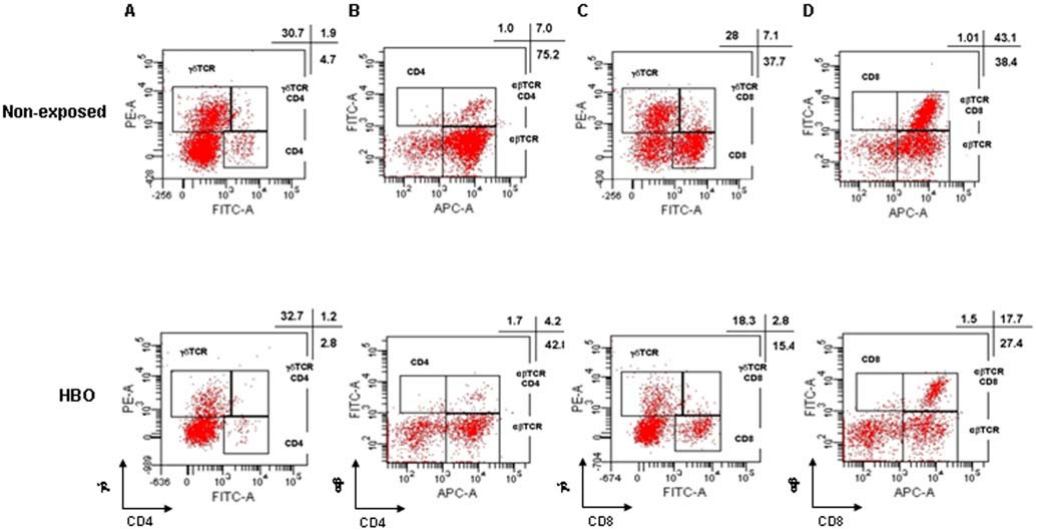
Reduced brain-sequestered T lymphocytes in PbA-infected mice exposed to HBO treatment. Flow cytometric analyses were done on γδ and αβ CD4^+^ and CD8^+^ T cells sequestered in mice brains (a pool of 4–5 mice per group) collected on day 7 after PbA infection between the groups regardless of exposure to HBO conditions. Pressurized oxygen therapy reduced the percentage of all cellular subsets, but mainly αβ CD8^+^ T cells. Representative dot blots of (A) γδ and CD4, (B) αβ and CD4, (C) γδ and CD8, (D) αβ and CD8 double staining.

Next, we asked whether the neuroprotective effect of the pressurized oxygen therapy could be associated to the percentage of γδ and αβ T lymphocytes sequestered in mice brains collected on day 7 p.i. (Figure 5). As compared with brains of non-exposed animals, HBO treatment reduced about 1.6 fold the percentage of both γδ (1.9 vs. 1.2%) and αβ (7.0 vs. 4.2%) CD4^+^ T cells between the pools of mice of these two groups (Figure 5A–B). However, a more pronounced decline, about 2.5 fold, was observed on the percentage of both γδ (7.1 vs. 2.8%) and αβ (43.1 vs. 17.7%) CD8^+^ T lymphocytes in the mice exposed to HBO in contrast to the non-exposed animals (Figure 5C–D). Taken together, our data demonstrate that HBO's neuroprotective effect is related to the reduction of the T cells sequestered in mice brains; and corroborate with existing literature, in which T lymphocytes, mainly αβ CD8^+^ T cells, are implicated in CM pathology [14], [37] No immunolabeling was detected on T lymphocytes in the absence of mAbs (data not shown).

### HBO effects on severe ECM symptoms

**Figure 6:**
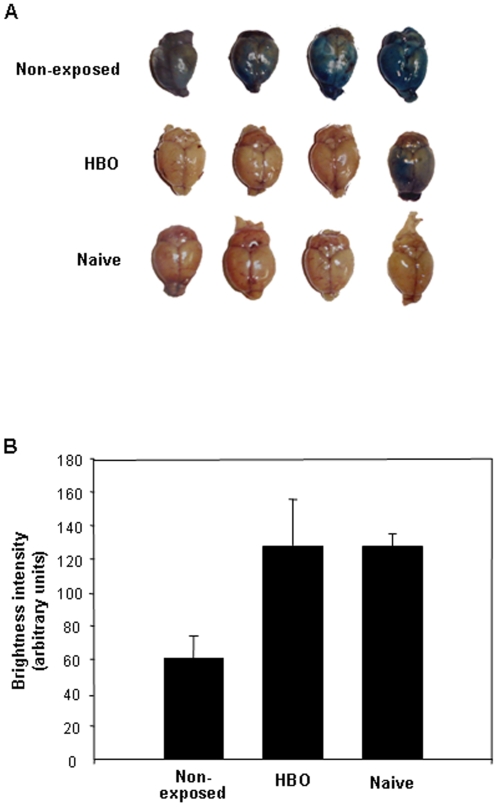
HBO preserves integrity of the blood-brain barrier in PbA-infected mice. Four PbA-infected mice, representative of each group (n = 8) exposed or not to HBO treatment (100% O_2_, 3.0 ATA, 1 hour per day), received i.v. injections of 1% Evans Blue solution early on day 7 p.i.. (A) One hour after Evans Blue injection, brains of naïve animals, PbA-infected mice and HBO-treated PbA-infected mice were collected and photographed (n = 4 of each group). (B) The BBB dysfunction of naïve mice or PbA-infected animals, regardless of submission to hyperbaric conditions, was determined by brain staining quantification with the aid of the ImageJ™ software (n = 4 of each group). HBO significantly reduced (*P*<0.005) the staining in the brains of infected-mice in comparison to non-treated animals. No statistical difference (*P*>0.05) was noticed between naïve and HBO-treated infected mice and brains collected from non-treated infected mice were significantly (*P*<0.005) darker than naïve animals. Results are expressed as the mean of brightness intensity of each delimited brain area of six mice±standard deviation.

Severe hypothermia and dysfunction of the BBB are common features in ECM [35]. To investigate whether HBO therapy could improve poor ECM outcomes, we measured the corporal temperature of PbA-infected mice daily regardless of exposure to pressurized oxygen in the same conditions as the 11-day exposure protocol. Unlike in the case of non-exposed mice, HBO therapy significantly prevented (*P*<0.001) hypothermia in mice from day 6 p.i., when severe neurological signs were evident in most of the animals (data not shown). Then, by injecting Evans Blue solution, we analyzed and quantified the BBB integrity in HBO exposed and non-exposed animals and in naïve animals early on day 7 p.i.. One hour after Evans Blue injection, mice brains were collected and photographed. As seen in Figure 6A, brains collected from non-exposed mice were darker than those of HBO treated animals due to a high incorporation of Evans Blue in the brain tissue as a consequence of BBB destruction [26]. As expected, we did not observe any staining in naïve mice brains. To quantify the Evans Blue staining and, in turn the BBB integrity, we measured the light intensity in naïve animals and infected mice brains submitted or not to pressurized oxygen. According to Figure 6B, HBO therapy significantly reduced (*P*<0.005) the brain staining in treated mice. Moreover, when we compared the Evans Blue staining in naïve and PbA-infected animals that received HBO treatment, no significant difference was observed (*P*>0.05). As expected, a statistical difference in light intensity levels was observed between naïve mice and non-exposed infected animals (*P*<0.005). Collectively, these data clearly demonstrate that HBO prevents temperature drops and BBB dysfunction.

## Discussion

In the present study, we show that HBO therapy (100% O_2_, 3.0 ATA) is capable of partially protecting PbA-infected mice against CM and delaying CM-specific neurological signs (Figures 1 and 3). These observations demonstrate for the first time that pressurized oxygen therapy under hyperbaric conditions well-tolerated in humans and animals can prevent CM clinical outcomes, including death.

In an experimental rat model of brain trauma, recent studies have shown that HBO has a neuroprotective effect against focal cerebral ischemia, especially when initiated within the first 6 hours [38]. HBO was thus found to reduce BBB damage, prevent apoptosis and maintain lipid oxidation levels stable [39]–[42]. HBO's neuroprotection was also observed in neonatal rats after the induction of the ischemic process [43]. Rabbits exposed to pressurized oxygen for 90 min during 3 consecutive days presented a significant reduction in the edema area of the brain and cerebral necrosis [44]. In addition, the preservation of BBB, the reduction in HIF-1α levels, and decreased apoptosis and neuronal damage were observed in a rat model for subarachnoid hemorrhage after exposure to HBO [45]. In humans, exposure of thirty-seven brain-injured patients to sixty minutes of HBO treatment every 24 hours increased the cerebral metabolic oxygen rate and reduced cerebrospinal lactate levels [46]. In another study, 10 out of 22 patients with cerebral infarction presented an amelioration of their motor function, while 7 of these patients experienced improved revascularization after pressurized oxygen sessions [47].

When comparing exposed animals with non-exposed animals, we noticed a significant reduction on the parasitemia levels of PbA-infected mice exposed to HBO (11-day exposure protocol) during infection (4–6 p.i.; Figure 1B). PbNK-65-infected mice exposed to HBO in the same conditions also presented a significant reduction of their parasite burden on day 4–13 p.i. (Figure 1C). These findings are in line with a recent study in which daily sessions of 100% pressurized oxygen at 2.5 ATA significantly reduced the size of *Leishmania amazonensis* induced lesions and the parasite development in infected mice [36]. Nevertheless, as in ECM parasites in the brain are necessary, but not sufficient, to neurological symptoms appearing [15], the lack of correlation between survival and the reduction of parasitemia levels, measured daily until the death of PbA-infected animals exposed to HBO, might be related to the fact that parasitemia levels probably do not determine the parasite load in the brain. Indeed, methods aimed at inducing protection against ECM often do not reduce parasitemia levels [48].

Also, direct exposure to HBO for up to 6 hours observed in our *in vitro* analyses was not harmful to normal or PbA-infected erythrocytes (data not shown and Figure 2), differing from previous studies where direct exposure of *L. amazonensis* promastigotes to HBO for up to 6 hours significantly decreased parasite viability [32]. However, as it is assumed that HBO increases the levels of reactive oxygen intermediates (ROI) [49], we believe that the disparity of these two protozoan parasites in terms of HBO susceptibility might be linked to differential killing mediated by reactive oxygen intermediates (ROI). In fact, it has been shown that *Leishmania* parasite killing is sensitive to ROI, whereas PbA-infected erythrocytes are resistant to killing by ROI, even at supraphysiological doses, and ROI are not essential for controlling *Plasmodium* sp. parasitemia [50]–[52].

We have also shown that the neuroprotective effects of daily hyperbaric sessions rely on the combination of hyperoxia and pressure at 3.0 ATA (Figure 1A), as ECM-specific mortality of PbA-infected mice submitted to 100% oxygen pressurized at 1.0 ATA did not differ significantly from the non-exposed animals (Figure 3B). In an experimental model for cerebral ischemia, HBO neuroprotection was not achieved in animals submitted to pure oxygen at only 1.0 ATA [39], [40], and human stimulated monocyte-macrophages cultured in hyperoxia did not present changes in their cytokine expression levels [23]. More importantly, in a study of 12 CM comatose patients who breathed 95% oxygen, no improvement in the consciousness levels were observed in any of the individuals [7].

Brain macrophages from adults and children who died of CM had higher levels of immunological markers that are normally not upregulated [9], such as IFN-γ, IL-1β, IL-10 and TNF-α [10], [11], [16] neuroprotection in ECM is often associated with the reduction of IFN-γ, and TNF-α levels [53]–[55]. IL-10 is higher in severe malaria patients from different regions despite the fact that CM individuals presented lower levels of IL-10 in comparison to the non-cerebral malaria group [16], [56] Furthermore, CD8^+^ αβ T cells migrating to the brain have been implicated in cytotoxicity and BBB disruption, thus contributing to ECM mortality [14], [15]. Here, we showed that HBO therapy reduced IFN-γ, TNF-α and IL-10 mRNA expression levels in the brain and the percentage of brain-sequestered CD4^+^ and CD8^+^ γδ and αβ T lymphocytes (Figures 4–5). Moreover, the reduction in the IL-10 levels in PbA-infected mice exposed to HBO might be associated with the decrease in expression of IFN-γ and TNF-α. These data are in line with the fact that pressurized oxygen is able to inhibit synthesis of cytokines, such as TNF-α and IFN-γ, T lymphocyte proliferation, decrease the migration of immunocompetent cells and improve tissue transplantation by down-regulating lymphoid system functions [19], [22], [23], [28], [57], [58].

Finally, when we assessed the HBO effects on cerebral outcomes, we noticed a significant reduction in hypothermia (data not shown) and in the BBB breakdown (Figure 6) in mice exposed to pressurized oxygen. This corroborates previous findings where HBO (100% O_2_, 2.8–3.0 ATA) prevented BBB permeability and functionality in animals submitted to a brain injury [31], [36]. Based on these observations, it is plausible to assume that HBO prevents BBB breakdown and then avoids vascular leakage by down-regulating the inflammatory immune response in ECM, but mainly, by reducing the percentage of brain-sequestered CD8^+^ T lymphocytes [10]. Therefore, we cannot rule out that other mechanisms are also involved in HBO neuroprotective effects in ECM, as HBO also inhibits ICAM-1 expression and neuronal apoptosis and upregulates the expression of vascular endothelial growth factor (VEGF), which is involved in angiogenesis in human endothelial cells [22], [23], [25], [28], [59]. Also, HBO led to an increase in the brain levels of nitric oxide (NO) [60], a molecule that contributes to protection against ECM [61].

In summary, we have presented evidence of the beneficial effects induced by HBO therapy against ECM. We also demonstrated that the administration of pressurized oxygen down- regulates IFN-γ, TNF-α and IL-10 cytokine expression and the migration to the brain of T lymphocytes, preventing BBB breakdown and severe mice hypothermia without directly affecting iRBC viability and infectivity. Since complementary therapies such as steroids, sodium bicarbonate and heparin are deleterious in CM, and treatment with an anti-TNF-α monoclonal can worsen neurological symptoms [62]. The data presented here create promising perspectives for further investigation of additional HBO's neuroprotective mechanisms and to consider it as a new supportive therapy that could act alone or in association with conventional treatment or with recently discovered neuroprotective or anti-inflammatory molecules to improve poor CM outcomes [63], [64].

## Supporting Information

Table S1 (0.01 MB PDF)
