# Supplementary material for: Hyperbaric Oxygen Prevents Early Death Caused by Experimental Cerebral Malaria
Source: PLoS One. 2008 Sep 4;3(9):e3126. doi: 10.1371/journal.pone.0003126 (PMC2518956; doi:10.1371/journal.pone.0003126)
Supplement: Table S1 — (0.01 MB PDF) [file pone.0003126.s001.pdf]

Table S1. Lack of association between parasitemia levels and neuroprotection in HBO[a] exposed animals infected with PbA[b].

Mice	Inhibition[c] (%) Day 4	Inhibition (%) Day 5	Inhibition (%) Day 6	CM neurological symptoms[d]
#1	44,31	35,06	9,53	No
#2	5,57	12.74	23,69	Yes
#3	62,71	2,59	15,82	Yes
#4	18,64	13,75	12,67	No
#5	-11,38	2,59	19,76	No
#6	-28,33	4,62	22,9	No
#7	22,52	12,74	26,05	No
#8	22,52	19.84	11,10	Yes
#9	63,68	6,65	25,26	Yes
#10	22,52	29,99	-36,99	Yes

[a] Animals were exposed to the 11-day exposure protocol .

[b] Mice were infected i.p. with 10^6 iRBC of *P. berghei* ANKA.

[c] Inhibition of parasitemia relative to non-exposed animals.

[d] All animals that presented CM neurological symptoms died on day 6-10 p.i..
